# Young species of cupuladriid bryozoans occupied new Caribbean habitats faster than old species

**DOI:** 10.1038/s41598-018-30670-9

**Published:** 2018-08-15

**Authors:** Aaron O’Dea, Brigida De Gracia, Blanca Figuerola, Santosh Jagadeeshan

**Affiliations:** 1 Smithsonian Tropical Research Institute, Box 0843-03092 Balboa, Republic of Panama; 2 Department of Physiology, University of Saskatchewan, 107 Wiggins road, Saskatoon, Canada

## Abstract

The breadth of habitat occupied by a species, and the rate at which a species can expand into new habitats has important ecological and evolutionary consequences. Here we explore when extant species of free-living cupuladriid bryozoans expanded into new benthic Caribbean habitats that emerged during the final stages of formation of the Isthmus of Panama. Habitat breadth was estimated using the abundances of over 90,000 colonies in ten cupuladriid species, along with the ecological and sedimentary characteristics of the samples in which they occurred. Data reveal that all species expanded their habitat breadths during the last 6 Myr, but did so at a different tempo. ‘Young’ species - those that originated after 5 Ma - expanded relatively quickly, whereas ‘old’ species - those that originated before 9 Ma - took a further 2 Myr to achieve a comparable level of expansion. We propose that, like invasive species, young species are less restrained when expanding their habitat breadths compared to older well-established species. Understanding the mechanism causing this restraint requires further research.

## Introduction

Species habitat breadth, or the suite of environments or resources that a species utilizes^1,2^, has important implications for the ecology and evolution of a species and its communities^3,4^. For example, species with wide habitat breadths (otherwise known as niche generalists) have larger geographic ranges^5^ resulting in lower probabilities of extinction^6–9^, and habitat breadth will dictate a species’ response to human-driven changes such as habitat degradation and fragmentation, and climate warming^10^.

The habitat breadth of a species is a consequence of its environmental tolerances^11^, the strength of interspecific competition^12–14^ and the availability of appropriate habitat within the geographic range of the species’ dispersal ability^15^. This is akin to the association-based niche breadth concept^3^, otherwise known as the environmental realised niche, climatic niche, Grinnellian niche or scenopoetic ecological niche^16^. Much work has explored the role that niche breadth has on fitness, population structure, diversification, and cladogenesis^3^. However, only a few studies have explored stasis and change in the breadth of habitat of species over their age-ranges using the fossil record. High quality fossil records based on replicated samples with stratigraphic resolutions of less than half a million years (Myr) can provide insights into the stability and evolution of habitat breadths within taxa over times of biogeographic and environmental change^17–21^.

Slow formation of the Isthmus of Panama over ~25 Myr^22^ constricted and then severed the seaway connection between the Tropical Eastern Pacific and the Southwest Caribbean (SWC), culminating in the formation of the Isthmus in the late Pliocene^23,24^. The shoaling Isthmus blocked the mostly eastwardly currents that caused upwelling waters to diminish in the SWC^25^, shutting-off a major supply of dissolved nutrients^25,26^ and causing Caribbean planktonic primary productivity to decline^27,28^. This major oceanographic reorganisation and resultant shifts in environment and coastal ecology led to an expansion in the types of benthic shelf habitats in the SWC. Before ~4 Ma, the SWC was dominated by upwelling waters and terrestrial-derived, muddy, organic-rich sediments, low in carbonate and little to no coral or coralline algae^25,29,30^. After ~4 Ma coral reefs flourished and carbonate deposition increased rapidly across the Caribbean, yet the muddy, organic-rich and low-carbonate sediments continued to exist in tandem. As a result, a wider gamut of habitat types existed in a ‘patchwork’ setting after 4 Ma to the present day^25,29^. This expansion of the types of habitats available to benthic organisms is thought to have played an important role in the diversification of Caribbean marine life during the Plio-Pleistocene^25,31–34^.

**Figure 1:**
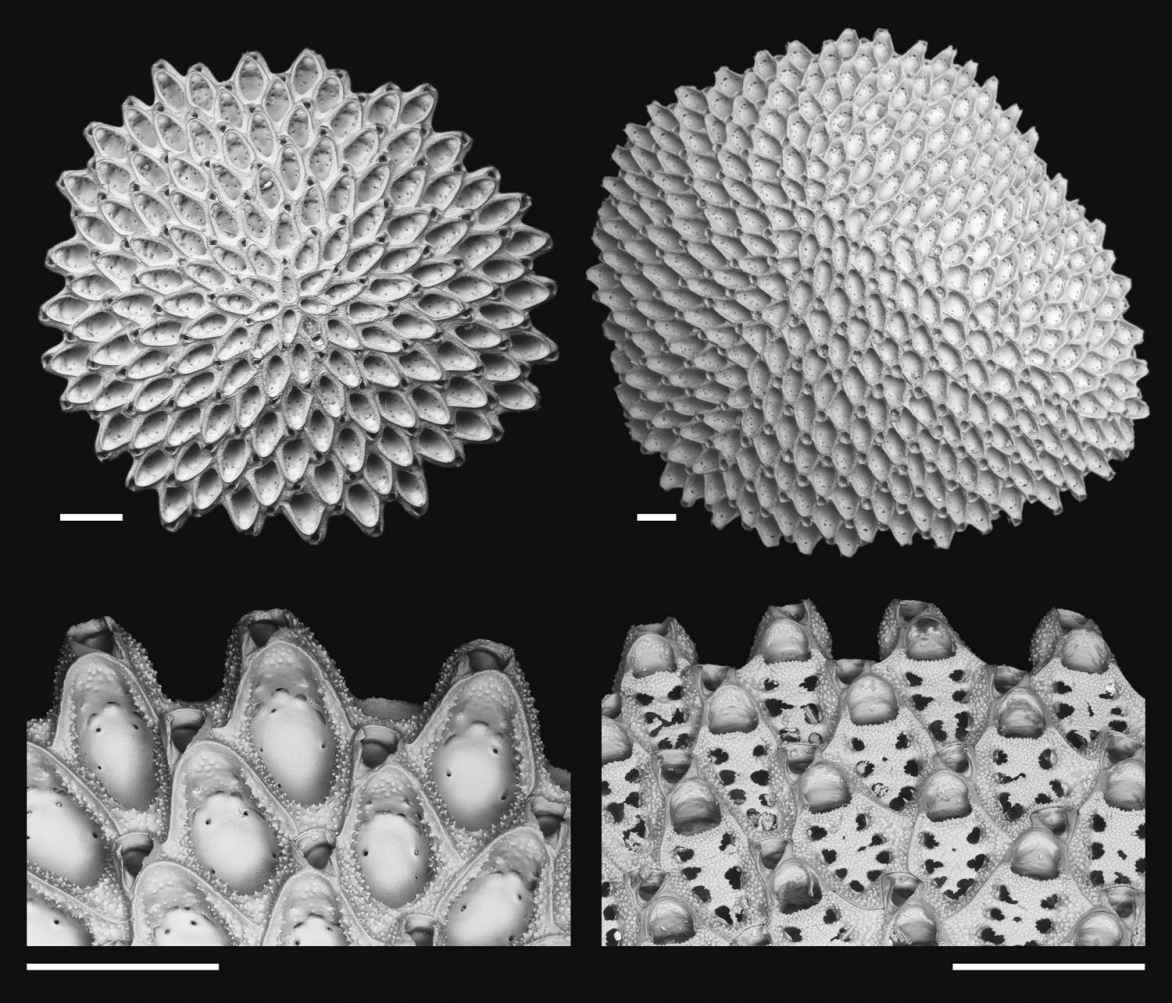
Examples of cupuladriid bryozoans and their morphology. Top, whole colonies of *Cupuladria biporosa* showing typical disc-like colony form with a sexually-produced colony (left) and a clonal colony (right). Bottom, marginal autozooids and vibracula of *C. biporosa* (left) and *Discoporella bocasdeltoroensis* (right). Scale bar = 500 µm. Images courtesy of Paul. D. Taylor.

In this study, we use an exhaustive collection of fossil and Recent free-living cupuladriid bryozoans (Fig. 1) to explore how and when the habitat breadths of species responded to the expansion of habitat types over the last 6 Myr in the SWC. Cupuladriid bryoozans are well-suited for this investigation because (a) they are abundant and fairly diverse^35^, (b) zooidal and colony characters permit robust identification to species^36,37^, (c) different species adopt dissimilar colony and zooidal morphologies which dictate how colonies interact with the benthos, move over and through sediments, and resist burial^38^, and presumably because of this (d) distributions of modern cupuladriid species are partitioned along sedimentological and biotic variables of the benthos variables^38,39^ and such variables can can be measured in the fossil record. We measure habitat breadth of cupuladriid species using the relative and sample intensity-corrected abundances of colonies along sedimentological and biotic gradients of the benthos in which they are found. Our findings suggest that habitat breadths of cupuladriid species are considerably dynamic over time and that the ability to take advantage of new habitat is defined by the age of the species.

## Results

**Figure 2:**
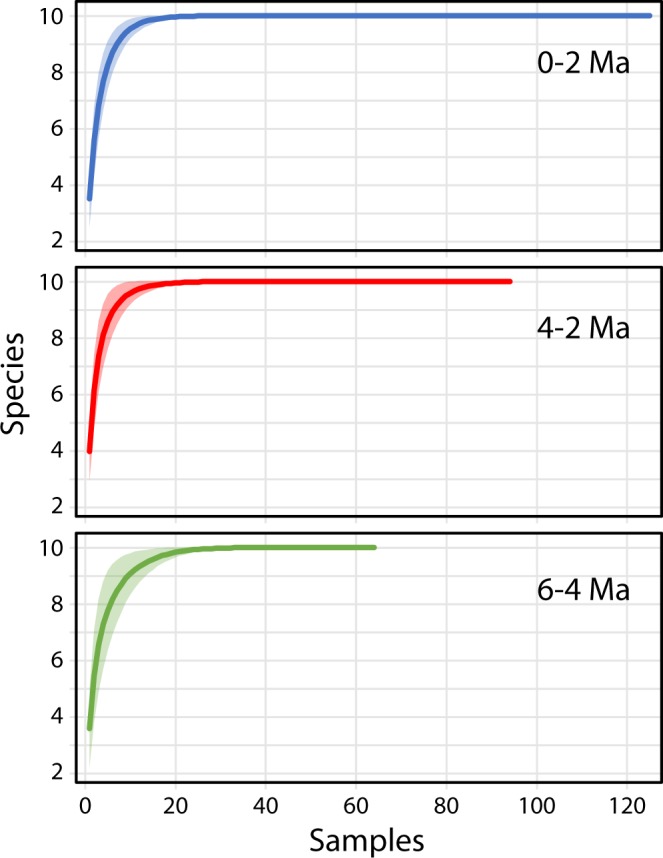
Rarefaction curves for the ten commonest cupuladriid species from fossil and Recent samples in the southwestern Caribbean grouped in three time-bins. Shaded area represent 95% Confidence Intervals. Calculated using EstimateS^87^.

**Figure 3:**
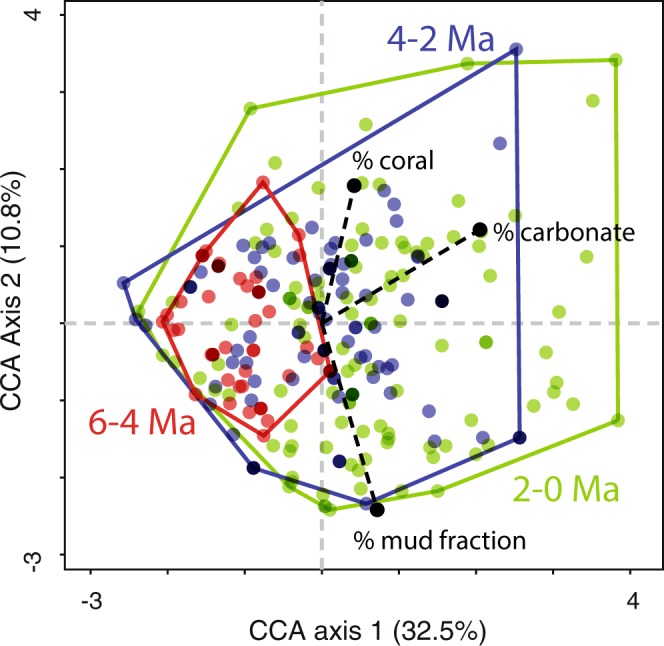
Changes in ecological space of cupuladriid bryozoans over the last 6 Myr in the southwestern Caribbean. Canonical Correspondence Analysis (CCA)^40,85^ of sample scores (dots) based on the relative abundances of species of cupuladriid colonies in bulk samples (fossil) and dredges (Recent). The CCA is constrained by the variables % calcium carbonate, % mud and % coral in samples, all of which expanded after ~4 Ma. Samples are grouped into 2 Myr bins.

**Figure 4:**
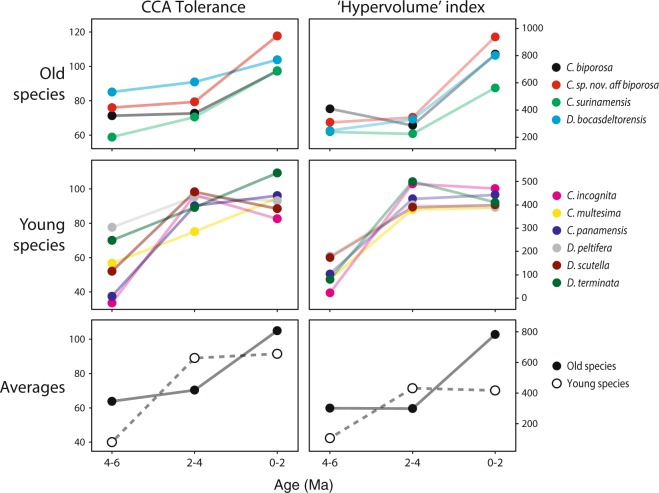
Expansion of habitat breadths of cupuladriid species in the southwestern Caribbean over the last 6 myr. Estimates of habitat breadth are split into old species (top), young species (middle) and averages of old and young species (bottom). Old species originated before 8 Ma and young species after 5 Ma. We calculated two estimates of cupuladriid habitat breadth - CCA Tolerances (aka RMSTOL^40,85^) (left) and *Hypervolume index* (right).

Our analysis of over 90,000 colonies from ten extant species of cupuladriid bryozoans comes from a well-sampled collection of Recent and fossil cupuladriids (Table 1, Fig. 2). Distributions of % carbonate, % coral and % mud in the same samples from where the cupuladriids were collected reveals all these variables expanded after 4 Ma^25^ (see Supplementary Fig. S1). Canonical correspondence analysis (CCA), based upon the relative abundances of cupuladriid species in samples and constrained by the same environmental variables, show that time-binned-species scores expanded substantially after 4 Ma (Fig. 3). We estimate the habitat breadth of each cupuladriid species in each of the three time bins using a *Hypervolume index* and Tolerance values from CCA^40^, both of which are independent of sampling intensity or sample size (see material and methods for further information). These two measures reveal similar patterns through time, and suggest that all ten cupuladriid species increased their habitat breadths substantially over the last 6 Myr (Fig. 4). The timing of this expansion was, however, different in ‘old’ and ‘young’ species. Young species that originated after 5 Ma expanded their habitat breadths between 4 and 2 Ma whereas well-established species older than 8 Ma expanded their habitat breadths after 2 Ma.

**Table 1:** Numbers of colonies of the 10 commonest cupuladriid species used in this study in samples over three time-bins.

Species	FO (Ma)	Age bins (Ma)	2-0	4-2	6-4	Totals
		# Samples	125	94	64	283
*C. biporosa*	10.15	*Old*	4437	7413	3094	14944
*C. sp. nov. aff biporosa*	12.6	*Old*	7751	4000	3798	15549
*C. incognita*	4.25	*Young*	8635	1441	43	10119
*C. multesima*	4.25	*Young*	1426	661	40	2127
*C. panamensis*	4.25	*Young*	1652	2883	260	4795
*C. surinamensis*	9	*Old*	19630	2864	513	23007
*D. bocasdeltoroensis*	12.6	*Old*	644	241	592	1477
*D. peltifera*	4.25	*Young*	1603	6633	2019	10255
*D. scutella*	4.25	*Young*	1732	4262	776	6770
*D. terminus*	4.25	*Young*	1052	315	125	1492
		**Total**	48562	30713	11260	90535

First occurrences (FO) of species is also listed and the number of samples per time bin. Total colonies analysed = 90,535. Total samples = 283 (195 fossil and 88 Recent). Note 0–2 Ma time bin includes 88 Recent dredge and 37 fossil bulk samples. FO’s derived from O’Dea and Jackson^52^.

## Discussion

Evidence suggests that some plants and animals maintain static habitat breadths over geological time scales. Beech trees in Europe, for example, moved south and north in sync with growing then retreating glaciers during the Pleistocene^41^, and similar patterns of ‘habitat-tracking’^42^ have been well-documented in many different groups, especially under times of rapid environmental fluctuations^43,44^. Fixed habitat breadths through the life trajectory of species, otherwise known as environmental niche stasis, is an assumption of many evolutionary models and most species distribution models used when predicting future responses^45,46^. Our findings show that habitat breadths of cupuladriid bryozoans can be dynamic over a species’ age-range, and that older species may be more restrained in their ability to expand into new habitats.

This finding may appear contradictory because geographic range is usually a good predictor of habitat breadth, and young species, whose geographic ranges are narrow, should therefore have smaller habitat breadths compared to well-established, widely-distributed and abundant older species. Nevertheless, young species have been shown to be able to rapidly increase their geographic ranges and abundances following origination^47,48^ implying that habitat breadths could also exhibit similar spatio-temporal patterns. Ecological divergence can also be rapid when closely related (i.e. young) sister species are sympatric^49,50^. And while older species may be more widespread, their well-established, large populations may be less able to escape from established roles and defined environmental tolerances because of high gene flow. This inability to take advantage of newly available habitat is termed niche conservatism^15,51^ caused by higher fitness of individuals in the original source habitat compared to the fitness of individuals that spread into new habitats^45^. Thus, while species with larger populations may have a greater potential to exploit new habitat by virtue of being widespread, they may be limited in doing so. Our data support this scenario, and it is interesting to note that estimated habitat breadths of older species, while slower to respond initially, eventually overtook their younger congenerics to achieve wider niche breadths in the last 2 Myr (Fig. 4).

There are alternative explanations for the patterns observed. One is that expansion into new habitats could have been restricted in old species because of cumulative limiting interactions with other biota. Theory suggests that new species will experience relatively lower levels of competition and other potentially limiting biotic interactions such as parasitism, and may therefore be less fettered relative to older species. This goes some way to explain why invasive species can often expand both their realised and fundamental niches in their invasive ranges^3^. In the SWC, competition from other filter feeding organisms may have declined 4-2 Ma when widespread extinction of benthic marine taxa resulted in a loss of 30–100% of benthic mollusc, bryozoan and coral species in the Caribbean Plio-Pleistocene^24,25,32,52–55^. Although cupuladriid species do partition their habitats between species, there is much overlap in the habitat breadths of cupuladriid species^38^, most likely because competition is diffuse in soft-bottomed shelf environments. It therefore seems unlikely that competition for either space or food would have declined in shelf habitats during the regional extinction, suggesting that competition may not have been a mechanism for the patterns observed.

Two further potential explanatory mechanisms that deserve mentioning relate to the reproductive life histories of the cupuladriid bryozoan species themselves. First, clonal reproduction is the dominant mode in ‘old’ species whilst in ‘young’ species it is sexual^52^. Both theoretical and experimental studies suggest that sexual populations are able to adapt more rapidly to new and variable environments^56–58^. An important reason is that sex can restructure allelic combinations and epistatic interaction in genomes, thus generating more variability in fitness, which should allow populations to rapidly exploit new niches or adapt to changing environments^57,59^. Thus, younger cupuladriid species may have had a higher propensity of adaptation given their higher rates of sexual reproduction. One approach to explore this hypothesis would be to observe if the taxa that reproduce only sexually (e.g. molluscs) also differ in their rates of habitat breadth in young and old lineages.

The second mechanism relates to dispersal ability, which is predicted to be positively correlated with rate of range expansion in species^60^. Populations of cupuladriids that clone (i.e. predominantly older species) will have a lower dispersal ability than cupuladriids that reproduce sexually via larvae (i.e. the predominantly ‘young’ species). Could the new habitats simply have taken longer to colonise by older clonal species? This seems particularly unlikely given the length of time available (millions of years) and the fact that even heavily clonal species always have some level of sexual reproduction^52^. Moreover, habitat breadth is correlated with geographic spread presumably because wider tolerance facilitates successful dispersal, and not the other way around.

Our results suggest that younger species are less restrained in their ‘ability’ to take advantage of new habitat compared to older closely-related species, but the underlying mechanism remains unclear. One fundamental problem in interpreting these findings is that it is currently impossible to resolve if the observed expansion of habitat breadths by cupuladriid species represents a change in the fundamental or the realized niche^61^. A shift in the realised niche would implicate competition and other biotic interactions, suggesting that ‘old’ species are less competitive than ‘young’ species. Alternatively, shifts to the fundamental niche would suggest that young species may experience less niche conservatism than ‘older’ species, or invoke another evolutionary mechanism. One roadblock is that the fossil record is, by definition, the realised niche. Nevertheless, that realised niche should expand and contract in distinct ways at distinct times if the process(es) driving the patterns are intrinsic or extrinsic, biotic or abiotic. For example, Stigall^17^ explored niche evolution vs. niche stasis in Late Ordovician brachiopods. The study found remarkable stability in species’ niches during times of environmental stability, even though new species were originating, which should be expected to increase competition. Strong shifts in niches were observed only when sea levels began to fluctuate, implicating abiotic rather than biotic drivers in niche evolution. Further research like this that carefully explores the interactions of environmental change, biodiversity and habitat breadths, coupled with measures of the fundamental niche through the use of extant species with excellent fossil records should be insightful.

Our evidence supports the conclusion that like geographic range and abundances of a species, habitat breadth of a species can vary over the age-range of a species, as predicted given the three traits are often deeply interrelated on both long and short time scales^51,62^. However, the three traits are all too often considered intrinsic properties of a species that are invariable over a species’ age-range, and this is demonstrably not the case^17,47,48,63^. Unlike geographic range and abundance, the breadth of habitat of a species does not start and end at zero along a species’ trajectory. A newly formed species will be armed with most of the environmental tolerances of its immediate ancestor population (although presumably not its ancestor species); if habitat breadth started at zero, the species would not exist. As such, geographic range may only be a good predictor of habitat breadth of a species^1,5,6,64^ during the middle portion of a species’ age-range.

## Conclusions

Data support the conclusion that species’ habitat breadths can be dynamic over the age-range of a species and that the speed at which new habitats can be exploited can differ across even closely related species. We find evidence that the age of a species (amount of time since origination) may be negatively related to the tempo at which new habitats can be successfully colonised, with younger species being less fettered than their older congenerics because of stronger niche conservatism in more established populations. However, limitations of the fossil record and a poor understanding of the extent of competition in benthic soft-bottomed communities makes interpretation challenging.

## Material and Methods

### Cupuladriid bryozoans

The Cupuladriidae are a family of cheilostome bryozoans abundant in shelf habitats today across the tropics^35,39,65^ with an excellent fossil record^52,66,67^. Unlike most bryozoans, their disc-shaped colonies (Fig. 1) are free-living, resting on the surface of sandy or silty seafloors where their lophophores can filter feed^39,65^. They are able to use their setae to maneuver over the seafloor and dig themselves out if smothered in sediment^38,68^. This remarkable life mode appears to confer great advantage as they are often the only epibenthic filter feeders on the soft-bottomed tropical shelf habitats and can be found in densities of several thousand per square metre in such environments^35,69^. The reproduction of new colonies occurs by aclonal (presumably sexual) reproduction through the production of short-lived^39,65^ larvae that settle from the plankton to form new colonies, or by clonal fragmentation or fission of previously established colonies^66,67,70^. Rates of clonal reproduction vary greatly across species^35,66^, even when apparently closely related^52^. Cupuladriids are able to control the rate at which they clone through the robustness of their colony architecture. This can be as simple as creating thick or thinly calcified colonies, or more complex specialised approaches to induce self-cloning (e.g. auto-fragmentation and colony budding)^38,39,66,67,70,71^. This variation in reproductive life history approach is well-preserved in the skeletons of cupuladriids^67^, offering an unrivalled opportunity to directly measure the life histories of individuals, assemblages and species over deep time using the fossil record^52,66^.

### Sampling

We compiled the occurrences and abundances of the ten commonest species of extant cupuladriid bryozoans in the SWC over the past ~10 Myr (Table 1). Data were derived from a collection of approximately 150,000 fossil and Recent cupuladriid colonies^52^. Recent collections were made with dredge samples in shelf and shallow slope sediments along the Caribbean coast of Central America from 1995 to 2006. Fossil collections were made with bulk samples (ranging in mass from ~8–10 kg each) from fossiliferous Neogene shelf and shallow slope sediments on the Caribbean slopes of Panama and Costa Rica. See Coates *et al*.^72,73^ and Collins and Coates^74^ for details on geographic and stratigraphic distributions of Neogene fossiliferous sections in the SWC.

Fossiliferous bulk samples were disaggregated by soaking in Glauber’s Salt^75^. Both fossil bulk and dredge samples were sieved with a 2 mm mesh, dried, sorted and picked for cupuladriid colonies. Ages of fossil samples are median values of minimum and maximum ages based on microfossils from the sample or interpolated from ages of samples stratigraphically above and below^72,73,76–81^. Samples used in this study are the same as those reported in O’Dea and Jackson^52^; see therein for further information on collection methods, and ages of fossiliferous samples. Material is housed in the Smithsonian Institution National Museum of Natural History, USA and at the Naos Marine Laboratory, Smithsonian Tropical Research Institute, Panama.

### Taxonomy and ages of species

Cupuladriid colonies were classified using the rich morphological characters preserved in their calcium carbonate skeletons (Fig. 1) according to methods described in Herrera-Cubilla *et al*.^36,37^ which can effectively discriminate to species, as confirmed with molecular sequence data^31,36,37,82^. The ten extant cupuladriid species used in this study are those with the most abundant fossil records through the time of interest (Table 1)^52^.

First occurrences (FO) of species ranged from 12.6 to 4.25 Ma (Table 1). Species whose FO was before 8 Ma are considered ‘old’ and species whose FO was after 5 Ma are considered ‘young’. The quality of the marine fossil record in the SWC is excellent after 6 Ma and sampling intensifies after 6 Ma (Fig. 2). FOs of ‘old’ species are therefore likely to to be older whereas FOs of ‘young’ are likely to be robust. This is corroborated by estimated ages of diversification from molecular phylogenetic analysis^31^. For example, the two ‘old’ species *Cupuladria surinamensis* Cadée, 1975 and *C. biporosa* (Canu & Bassler, 1923) are estimated to have split in the middle Miocene (~15 ma), whereas ‘younger’ species such as *Discoporella scutella, D. peltifera*, and *C. incognita* are all estimated to have diverged in the Pliocene^31^. The close alignment of the FO’s with estimated ages of lineages from molecular phylogenetic analysis and the intensity of sampling, and the quality of the fossil record (Fig. 2) support our designations of ‘old’ and ‘young’ species.

### Estimating habitat breadths

Of the 24 extinct and extant species of cupuladriid reported in O’Dea and Jackson^52^ we estimated the habitat breadths of the ten commonest extant species (Table 1). We analysed a total of 90,535 colonies collected from 283 samples spanning the last 6 Ma (Table 1). Time bins were constructed as 6-4, 4-2 and 2-0 Ma, and samples assigned to time bins based upon their median age, as determined from the oldest and youngest age estimates from occurrences of planktonic foraminifera and nannoplankton taxa and magnetostratigraphy^72^.

Cupuladriid occurrences and distributions are tightly controlled by benthic habitat type^38,83,84^. We therefore describe the environmental conditions of samples in which cupuladriids occurred by focussing on sedimentary and benthic ecological characteristics, all of which were measured directly from the same samples, or adjacent samples from the same sections. Measurements made were % carbonate (loss of CaCO3 through HCL digestion) and % mud (<63 µm) fraction content of the samples and the % coral component of all skeletal weight in the samples. Percent carbonate and % mud fraction values were determined from the mean of triplicate analyses for Recent dredge samples, or individual analyses for fossil samples. Percent coral weight in total skeletal weight was calculated from samples by sieving at 2 mm and picking all coral material. Depth was measured for Recent samples and estimated for fossil samples using microfossil analysis^72,80^, and was used as a covariate in the CCA analysis. See^25^ for details on the collection of environmental measures and estimates.

Two techniques were used to estimate habitat breadths occupied by cupuladriid species in each time bin: Approach 1. *CCA Tolerance*. The root mean square standard deviation derived from the sample scores across the first four axes of a single Canonical Correspondence Analysis (CCA) (aka. Tolerance or RMSTOL^40^) has been frequently used as a measure of habitat breadth. CCA was performed using CANOCO^85^ based upon the relative abundances of time-binned-species in samples and constrained by the habitat variables described above for each sample. The RMSTOL value for each time-binned-species was used as an estimate of habitat breadth. For further information of the calculation of RMSTOL and its use as an estimate of habitat breadth see^40,86^. Approach 2. *Hypervolume index.* The product of the coefficient of variances (CV) of all time-binned species’ abundances along the three habitat variables. Species abundance distributions were corrected for varying sampling intensities along said gradients by creating 10 bins for each variable and dividing the abundance of each species in each bin by the total number of samples taken in each bin. CV’s were used instead of standard deviations to standardize variances for differences in the means of habitat variables. The resulting value is akin to Hutchinson’s hypervolume^61^.

The two estimates of habitat breadth are semi-independent; *CCA Tolerance* uses relative abundances of species in samples and the *Hypervolume Index* uses absolute abundances corrected for sampling intensity.

### Data availability

Raw data used in this study is available in the Online Supplementary Information.

## Electronic supplementary material

Online Supplementary Information

Supplementary Dataset 1
