# Supplementary material for: Young species of cupuladriid bryozoans occupied new Caribbean habitats faster than old species
Source: Sci Rep. 2018 Aug 15;8:12168. doi: 10.1038/s41598-018-30670-9 (PMC6093879; doi:10.1038/s41598-018-30670-9)
Supplement: Supplementary file 1 — Online Supplementary Information [file 41598_2018_30670_MOESM1_ESM.pdf]

Young species of cupuladriid bryozoans occupied new Caribbean habitats faster than old species

Aaron O'Dea[1]*, Brigida De Gracia[1], Blanca Figuerola[1], Santosh Jagadeeshan[2]

1. Smithsonian Tropical Research Institute, Box 0843-03092, Balboa, Republic of Panama
2. Department of Physiology, University of Saskatchewan, 107 Wiggins road, Canada

* = Corresponding author, email odeaa@si.edu

Online Supplementary Information

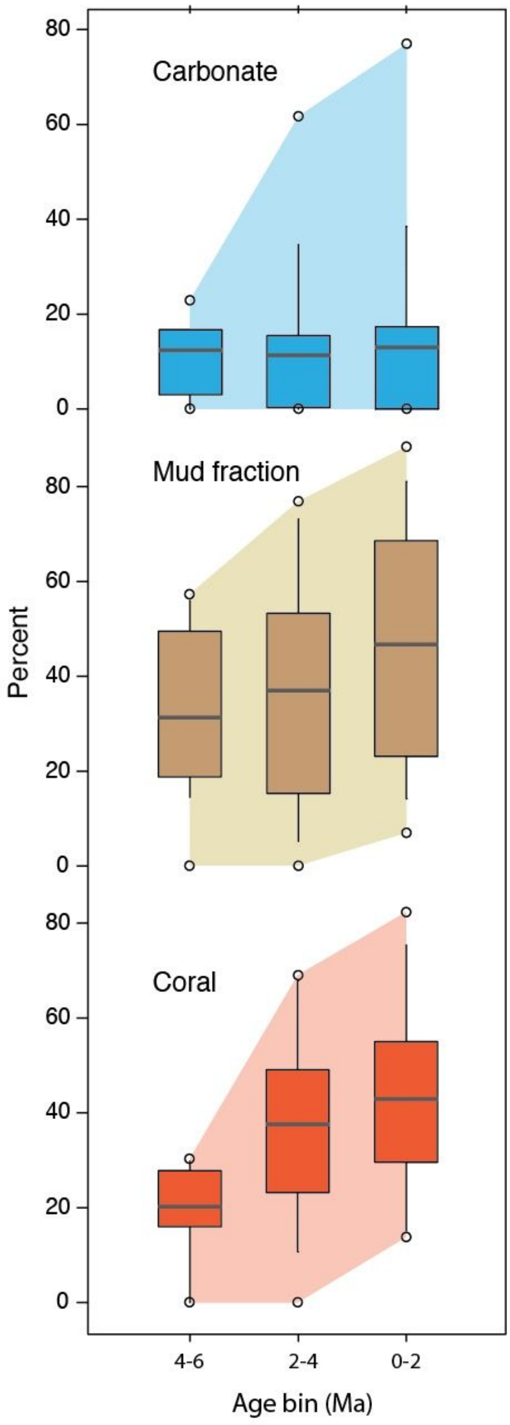

Supplementary Fig. S1. Expansion of benthic habitats in the southwestern Caribbean over the last 6 Myr. Variables measured include % carbonate and % mud (<63 µm) in sediments, and % coral in >2 mm in fraction. Boxes represent 25th and 75th percentiles, whiskers 10th and 90th percentiles, and dots 5th and 95th percentiles (shaded). Horizontal grey bars represent the mean. Data is from O'Dea et al.[1] and is also available in Online Supplementary Information.

1. O'Dea, A. *et al.* Environmental change preceded Caribbean extinction by 2 million years. *Proc. Natl. Acad. Sci. U. S. A.* **104,** 5501–5506 (2007).
